# The optoelectronic properties improvement of double perovskites Cs_2_SnI_6_ by anionic doping (F^−^)

**DOI:** 10.1038/s41598-022-04960-2

**Published:** 2022-01-18

**Authors:** Junsheng Wu, Zhuo Zhao, Yanwen Zhou

**Affiliations:** 1 School of Chemical Engineering, University of Science and Technology LiaoNing, Anshan, 114051 China; 2 Research Institute of Surface Engineering, University of Science and Technology LiaoNing, Anshan, 114051 China

**Keywords:** Solar cells, Optical materials, Energy

## Abstract

Tin-based perovskite material is the best choice to replace heavy metal element lead during the last several years. Cs_2_SnI_6_ with Sn^4+^ is a fascinating optoelectronic material, which is a more air-stable composite cesium tin halide peroxide variant from CsSnI_3_. However, the optoelectronic performance between N and P type of Cs_2_SnI_6_ varies considerably. Herein, we synthesized uniform Cs_2_SnI_6_ by modified two-step method, which thermal evaporated CsI firstly, and followed annealing in the SnI_4_ and I_2_ vapor at 150 °C resulted in uniform Cs_2_SnI_6_ films. SnF_4_ is used as a dopant source to improve the optoelectronic properties of Cs_2_SnI_6_ films. Results indicate that good crystallinity was obtained for all films and the doped films underwent a crystalline plane meritocracy transition. The doped films had a flat, non-porous morphology with large grains. The high transmittance of the doped films in the infrared region led to the avoidance of self-generated thermal decomposition. With the help of F^−^, the films became more conductive and had higher carrier mobility. DFT calculations showed that doping with F reduced the surface energy of (004), resulted in a preferred orientation transition in the crystal of Cs_2_SnI_6_. Fluorine doped double layer perovskite materials would have a broader application prospect.

## Introduction

In recent years, there has been extensive research into the emerging technologies of organic photovoltaics (OPVs) and perovskite solar cells (PSCs). Both OPVs and PSCs offer the potential advantages of being low cost, lightweight, flexibility and aesthetically pleasing^1^. While the commercialization of OPVs has recently been initiated, the younger but more efficient PSCs technology still needs to overcome some key issues, namely the toxicity of lead (Pb)^2,3^ and the mediocre stability of PSCs^4^, before it can enter the market. OPVs and PSCs still need to be further developed from the perspective of materials and equipment processing to improve their performance, reach the theoretical limit, improve their environmental stability, and replace their toxic components with fewer harmful substitutes.

Tin-based perovskite material is the best choice to replace heavy metal element lead. However, due to the inherently unstable Sn^2+^ of CsSnI_3_ perovskite, it is easily oxidized to Sn^4+^, and its stability is poor, so it has not been able to be manufactured and applied under air conditions^5^. In the previous work, we found Cs_2_SnI_6_ is a more air-stable composite cesium tin halide peroxide variant from CsSnI_3_^6^. The experimental results^7^ show that the intrinsic Cs_2_SnI_6_ is an N-type semiconductor with electron mobility up to 310 cm^2^ v^−1^ s^−1^. It is worth noting that when a certain amount of Sn^2+^ is doped, Cs_2_SnI_6_ is a P-type semiconductor, and its hole mobility is lower than the intrinsic electron mobility, which is 42 cm^2^ v^−1^ s^−1^. This also shows that Cs_2_SnI_6_ has dual carrier transport characteristics. In general, the carriers of semiconductor materials with lower mobility are more likely to compound, which in turn hinders the electron transport performance of the device. Therefore, it is worth exploring how to improve the performance of P-type Cs_2_SnI_6_ for application in the hole transport layer.

Doping is based on the artificial and controlled regulation of carrier density. The performance of semiconductor depends on the control of the type (P or N) and density of carriers (electrons or holes) in the device^8^. Therefore, it is a widely used method to dope any kind of impurities into the crystal lattice to control crystal growth and stability or adjust the photoelectric characteristics^9–11^. In the case of perovskites, X-site doping shows irreplaceable effects in modulating band gap and wavelength^12–16^. F^−^ is commonly added to tin-based perovskites precursor solutions, which reduces the oxidation of Sn^2+^ to Sn^4+^^5^. However, the effect of F^−^ on tin-based perovskites is not limited to their effect on this oxidation reaction. It is also of great benefit to the optoelectronic properties of tin-based perovskites. Consequently, we consider using F^−^ to modify the photoelectric performance of Cs_2_SnI_6_.

Herein, we synthesized Cs_2_SnI_6_ film by evaporating CsI firstly and annealing @150 °C for 1 h in SnI_4_ (SnF_4_) and I_2_ environment in a quartz tube furnace, which modified from Mitzi’s group^17^. This method enables the preparation of perovskites at low temperatures under atmospheric conditions, providing a new idea for the synthesis of stable and efficient perovskites. The structure and properties of Cs_2_SnI_6_ thin films with the amount of doping were observed, measured, and analyzed the influence of F^−^ doping. Meanwhile, to understand the underlying mechanism of performance change, we studied crystalline Cs_2_SnI_6_ over the amount of doped via density functional theory (DFT).

## Results and discussion

**Figure 1:**
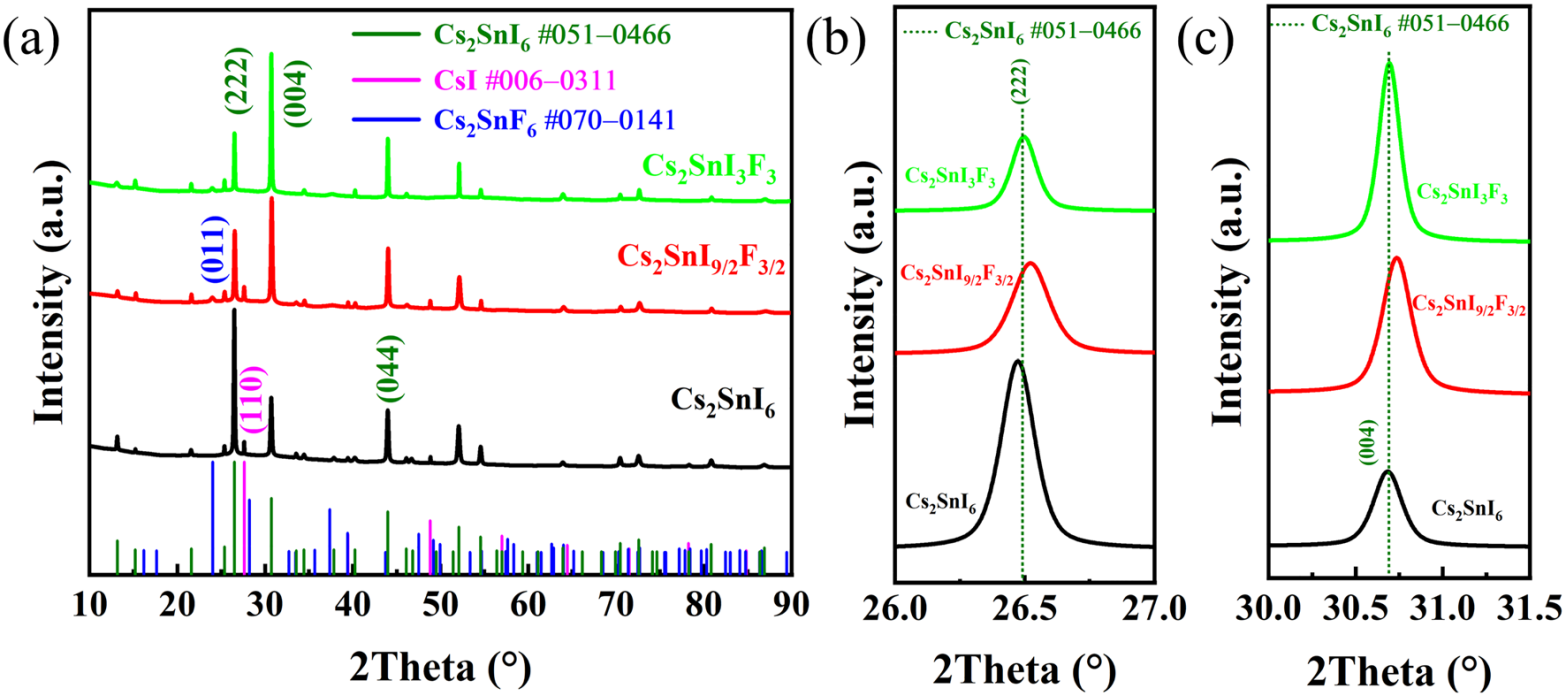
(**a**) XRD patterns of Cs_2_SnI_6_ films with F doped; (**b**) local enlarged (222) diffraction peak; (**c**) local enlarged (004) diffraction peak.

The phase structures of Cs_2_SnI_6_ films with F doped were characterized by XRD, and the results are shown in Fig. 1. Specifically, according to PDF #051-0466, the three strongest two-theta peaks located at 26.4731°, 30.6832°, and 43.9581°, corresponding to the (222), (004), and (044) diffraction planes of of Cs_2_SnI_6_, respectively^17^. Meanwhile, the well-matched peak of 27.5982°, assigned to CsI phase (110) plane (PDF #006-0311), was observed in Cs_2_SnI_6_ and Cs_2_SnI_9/2_F_3/2_ films. As predicted, the peak at 23.9748° was attributed to Cs_2_SnF_6_ phase (011) plane (PDF #070-0141) in F doped films. However, from the intensity of diffraction peaks, the main phase composition of the films was Cs_2_SnI_6_.

It is worth mentioning that the preferred orientation plane of Cs_2_SnI_6_ phase changed from (222) to (004) after doping, as shown in Fig. 1a. It can be seen from the local enlarged patterns (Fig. 1b, c) that as the doping amount increased, the intensity of the (222) diffraction peak decreased, and the FWHM increased. However, the intensity and FWHM of the (004) diffraction peak both gradually increased. In order to explain the differences of Cs_2_SnI_6_ (222) and (004) peaks on different F^−^ contents in this study, the diffraction peak offsets, FWHM, interplanar spacing offsets of the (222) and (004) peaks, the lattice constant and the volume of unit crystal on different F^−^ contents are compared and listed in Table 1. It can be seen from the periodic table that the ionic radius of F^−^ is smaller than that of I^−^. When F^−^ is doped into the Cs_2_SnI_6_ unit cell, the lattice constant is compressed. It can confirm this view by calculating the results of the volume. These changes are in full compliance with Prague's law. It further proved that F^−^ was successfully doped into Cs_2_SnI_6_. It is undeniable that after a large amount of F^−^ doping, a new phase was formed, which Cs_2_SnI_6_ was alloyed. Regarding the preferred orientation transition, it may be related to the change of the crystal plane energy and surface energy by the doping of F^−^. Surface/interfacial energy is the main factor influencing the formation and evolution of the film weave^18^. The higher the crystal planes' energy, the quicker the atomic accumulation rate, and then the faster the growth rate in the direction perpendicular to the crystal plane. There are two consequences of this: (1) The crystal grows rapidly along the direction perpendicular to the crystal plane; (2) The crystal plane disappears during the growth process. Therefore, we have performed first-principles calculations on the (222) and (004) crystal planes of Cs_2_SnI_6_, and we will discuss this issue in more detail in the part of calculation.

**Table 1:** XRD data of Cs_2_SnI_6_ films with F doped.

Sample	Cs_2_SnI_6_ phase (222)			Cs_2_SnI_6_ phase (004)			Lattice constant	The volume of unit crystal (Å^3^)
	∆θ(°)	FWHM(°)	∆d(Å)	∆θ(°)	FWHM(°)	∆d(Å)	a = b = c(Å)	
Cs_2_SnI_6_	− 0.0169	0.1071	0.00217	− 0.004	0.1347	0.00038	11.6475	1580.14
Cs_2_SnI_9/2_F_3/2_	0.0319	0.1715	− 0.00392	0.048	0.197	− 0.00443	11.6307	1573.31
Cs_2_SnI_3_F_3_	0.004	0.1451	− 0.00048	0.006	0.1647	− 0.00051	11.6424	1578.07

To explore the surface chemistry, the pristine and doped films were studied by X-ray photoelectron spectroscopy (XPS), which were estimated by curve fitting of the Cs 3d, Sn 3d, I 3d and F 1s spectra. The XPS spectra of these species are shown in Fig S1. These peaks were assigned according to the reference database^19^. All the curve-fitting analysis of XPS peaks for Cs, Sn, I and F demonstrate very identical spectra indicating the absence of multiple states of constituent elements. The peaks with binding energy of 723.22 and 737.14 eV can be attributed to the Cs 3d_5/2_ and Cs 3d_3/2_, respectively. It can be clearly seen that the spectra can be divided into distinct two peaks which can be attributed to I 3d_5/2_ (617.85 eV) and I 3d_3/2_ (629.33 eV), respectively. The XPS spectra for Sn showed a well symmetric characteristic peak at ~ 485.83 and 494.25 eV for Sn 3d that correspond to Sn^4+^ species. This result is consistent with earlier reports^20^. For doped films, the characteristic peaks at 684.42 eV (Fig S1d) were assigned for F 1 s that suggests the F^-^ state. The results of XPS indicate that these elements exist in the doped films as Cs^+^, Sn^4+^, I^−^and F^−^.

**Figure 2:**
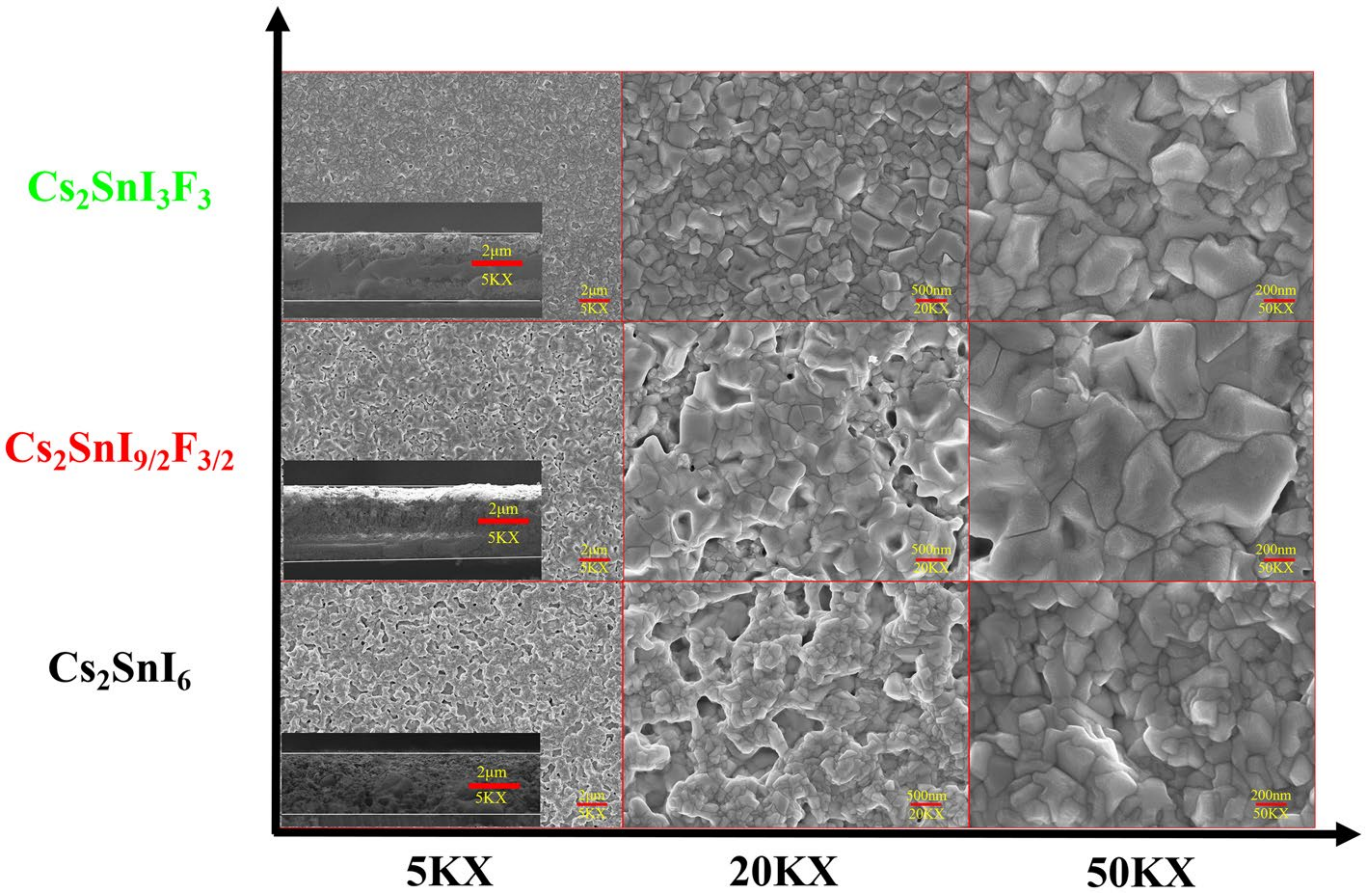
The top-view FE-SEM (field-emission scanning electron microscopy) images of Cs_2_SnI_6_ films with F doped (inset showed the cross-sectional image of respective films). Note: 5KX, 20KX and 50KX from left to right, Cs_2_SnI_6_, Cs_2_SnI_9/2_F_3/2_ and Cs_2_SnI_3_F_3_ from bottom to top.

The surface morphological structures of the Cs_2_SnI_6_ films with F doped are displayed in Fig. 2 and inset showed the cross-sectional image of respective films. The morphology of the Cs_2_SnI_6_ films on glass substrates were relatively flattening. However, holes were present in the films and decreased with increasing doping F^−^, eventually becoming almost invisible in Cs_2_SnI_3_F_3_. The appearance of the holes is due to the expansion of the crystal lattice during the chemical reaction between cesium iodide (CsI) and tin iodide (SnI_4_), releasing a lot of stress. This phenomenon is consistent with the results of Byungho Lee's experiments^21^. The grain size of the Cs_2_SnI_6_ films also became larger as doped F^−^ increased, see 50KX, most likely due to the aggregation of small grains. In previous works, the surface coverage of FASnI_3_ can be improved by adding F^−^ to reduce the pinhole and void forms present in pure perovskites films^22,23^ and even to obtain larger crystal sizes^24^. Furthermore, to confirm the elemental distribution, we also performed the elemental mapping for pristine and the doped films (Fig S2). It is found that Cs^+^, Sn^4+^, I^-^ and F^-^cations are evenly distributed in all the doped films. This further shows that the doping of F helps to obtain a film with a flat surface, no holes, and larger grains.

**Figure 3:**
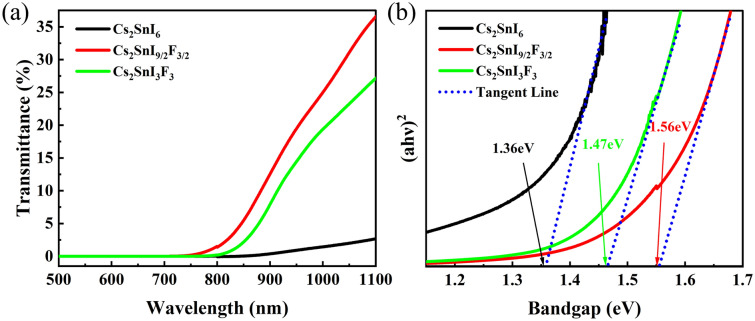
(**a**) Optical transmittance spectra of Cs_2_SnI_6_ films with F doped; (**b**) Tauc fit of the transmittance data assuming a direct bandgap for Cs_2_SnI_6_ films with F doped.

To investigate the optical properties of these Cs_2_SnI_6_ films, we performed the UV–VIS–NIR transmittance spectrum measurements within the range of 500–1100 nm, as shown in Fig. 3a. Obviously, there is a significant difference between the doped films and the undoped film. The intrinsic film absorbed almost all within the test range. However, F^-^doped films had higher transmittance in the infrared band. This effectively avoids the problem of self-decomposition of the perovskite material during application due to thermal effect of infrared radiation. The excellent optical properties of Cs_2_SnI_6_ films made up for this issue. The bandgaps of Cs_2_SnI_6_ films with doped F^−^ were also calculated by the UV–VIS–NIR transmittance spectrum and the plot of (αhv) versus photon energy (hv)^25^, as shown in Fig. 3b. The bandgaps of all the Cs_2_SnI_6_ films over doped F^-^ were 1.36 eV, 1.56 eV and 1.47 eV, separately. It is reported that Cs_2_SnI_6_ had a direct optical gap of 1.25–1.62 eV^7,17,26,27^. The results of the optical bandgap are similar to those of previous studies. It can be known from the DFT calculations of predecessors that the bandgap of Cs_2_SnF_6_ was 5.337 eV^28^, which is much larger than 1.3 eV of Cs_2_SnI_6_^29^. The results showed that the metal-halogen bond angle has an important influence on the band gap, which the bandgap decreases with the increase of the bond angle^30^. The minimum bond angle of F-Sn-F measured from the unit crystal of Cs_2_SnF_6_ was 87.591°, while that of I-Sn-I in Cs_2_SnI_6_ was 90°. F^−^ doping may induce increase of bandgap. What is surprising is that the band gap of Cs_2_SnI_3_F_3_ had become smaller. For semiconductors with the same crystal structure, small lattice constant means small interatomic distance, and hence, a strong electrostatic attraction. The band-gap represents the energy needed for bond-breaking, which reflects the strength of the attractive force. So, a direct consequence of decreasing the lattice constant is the increase in the energy gap. This is supported by the lattice constant in XRD (Table 1), which the lattice constant of Cs_2_SnI_9/2_F_3/2_ was smaller than that of Cs_2_SnI_3_F_3_. It is thus clear that F^−^ can adjust the bandgap of Cs_2_SnI_6_. In the future, by controlling the doping ratio of I–F, continuous modulation of the absorption of perovskite materials can be achieved.

The electrical properties and the thickness of the Cs_2_SnI_6_ films with doping F^−^ were shown in Table 2. All films were P-type semiconductor properties. In Table 2, it's also worth noting that the resistivities of Cs_2_SnI_6_ films with doped were an order of magnitude smaller than that of undoped film. And then the undoped Cs_2_SnI_6_ film exhibited the carrier densities of ~ 10^15^ cm^−3^, and the carrier density of Cs_2_SnI_6_ films remained almost the same order of magnitude with the increase of SnF_4_ content. At the same time, the carrier mobility of doped films is an order of magnitude greater than that of the undoped. The carrier concentrations were not significantly varied, while the carrier mobility of Cs_2_SnI_6_ for doped F exhibited the large varied, compared with Byungho Lee's experiments about ≈5 × 10^16^ cm^−3^ and 4–8 cm^2^ v^−1^ s^−1^^21^. These changes can be analyzed from the micro and macro perspectives, respectively. On one hand, F ions have a distortion effect on the crystal structure. The electronegativity of F is the largest in the periodic table. Therefore, more electrons were taken from Sn via F than I, the bond length of F-Sn was shorter, and the bond energy was higher^6^. On the other hand, F ions may promote crystal growth, and close to fewer defects. The lower porosity without visible crack formatted in the doped films from the SEM images (Fig. 2). In the crystal, the fewer defects (such as voids, grain boundaries, impurities, etc.), the less hindrance to the carrier, So the higher mobility is displayed in the films. In a word, the integrity of the lattice structure is poor, and there are many defects. The scattering and trapping of carriers are greatly enhanced. The carrier concentration and mobility are low, and the resistivity of the film is increased. The beneficial increase brought by F ions is the improvement of electrical properties.

**Table 2:** The electrical properties and thickness of of Cs_2_SnI_6_ films with doped F^−^.

Sample	Sheet resistance (Ω sq^−1^)	Resistivity (Ω·cm)	Carrier concentration (cm^−3^)	Carrier mobility (cm^2^ v^−1^ s^−1^)	Thickness (μm)
Cs_2_SnI_6_	7.07E+5	1.55E+2	1.51E+15	2.84E+1	2.24
Cs_2_SnI_9/2_F_3/2_	3.30E+5	7.26E+1	4.40E+14	1.95E+2	2.21
Cs_2_SnI_3_F_3_	8.80E+4	1.93E+1	1.67E+15	1.93E+2	2.83

The crystal structure was optimized to calculate the (222) and (004) crystal plane formation energy for Cs_2_SnI_6_. The crystalline surface formation energy^31^ is calculated as follows:

1 $$E_{f} = \left( {E_{slab} - NE_{bluk} } \right)/2A$$

where *E*_*f*_ is the formation energy of a crystal plane, *E*_*slab*_ and *E*_*bluk*_ are the total energies of the surface slab and the bulk unit cell, respectively, *N* is the number of atoms in a unit cell, *2* is forming two news along the Z-axis in the surface slab, and *A* is the surface area in the surface slab.

The calculated results are illustrated in Table 3. The lower the formation energy, the easier and more stable it is to form. There is a large variation in the formation energy after doping, especially in the (004) crystal plane. It is noteworthy that the formation energy increment of (004) is much larger than that of (222), which is 0.057 eV and − 0.178 eV, respectively. It can be seen that the growth rate of (004) crystal plane increased rapidly when doped with 1 F^−^, while the growth rate of (222) crystal plane slowed down. Then, at 25% or 50% doping, a change in crystal orientation is a natural consequence. The calculation results also prove the changing trend of the morphology of the thin films. After doping F, the pores of the films decreased, and the grains became larger.

**Table 3:** The calculated results of (222) and (004) crystal plane.

	(222) of Cs_2_SnI_6_	(222) of Cs_2_SnI_6_ with doped	(004) of Cs_2_SnI_6_	(004) of Cs_2_SnI_6_ with doped
*E*_*slab* (eV)_	− 22,440.277	− 21,475.320	− 3949.225	− 6507.373
*E*_*f* (eV)_	− 0.231	− 0.224	− 0.066	− 0.123

## Conclusion

In conclusion, we reported the double perovskite Cs_2_SnI_6_ film was successful prepared through a modified two-step method. Meanwhile, the effect of doped F^−^ on the optoelectronic properties of Cs_2_SnI_6_ films was also investigated. All the films revealed good crystallization as revealed by XRD. In particular, the (004) crystal plane is used as the preferred orientation of the F-doped films. In terms of film morphology, as the amount of doping increased, the surface tended to be flat and free of holes, and larger grain sizes were obtained. It is observed that the film analysis by XPS and EDX mapping indicates uniform composition in the film surface. Moreover, all films exhibited excellent light absorption in the visible range and the films with doped F^−^ increased transmission in the infrared region, which effectively avoids thermal-effect of infrared radiation. And then, the electrical properties of Cs_2_SnI_6_ have also been optimized. The resistivity of the films was reduced, while the carrier mobility was enhanced. These results may provide useful assistance for the application of Cs_2_SnI_6_ on HTM or LAM in solar cells. First-principles calculations confirm that doping with F changed the formation energy of the crystal plane. In the future, by controlling the doping ratio of I-F, continuous modulation of the absorption of perovskite materials, optimizing electrical performance, structure and morphology can be achieved. Therefore, we submit that doping F^−^ will be a sustainable study for future tin-based perovskite studies.

## Experimental details

### Materials

99.99% Cesium iodide (CsI, CAS No :7789-17-5), 99.99% Tin (IV) iodide (SnI_4_, CAS No: 7790-47-8), 99.99% Tin (IV) fluoride (SnF_4_, CAS No: 7783-62-2) were purchased from Advanced Election Technology Co., Ltd., Yingkou, China. 99.99% Iodine (I_2_, CAS No: 7553-56-2) was purchased from Aladdin Biochemical Technology Co., Ltd., Shanghai, China. 99.7% Acetone (Cas No: 67-64-1) and 99.7% ethanol (Cas No: 64-17-5) were purchased from Sinopharm Chemical Reagent Co., Ltd. China. Deionized water was filtered in the laboratory. All the reagents were of analytical grade and used as received.

### Synthesis of Cs_2_SnI_6_

**Figure 4:**
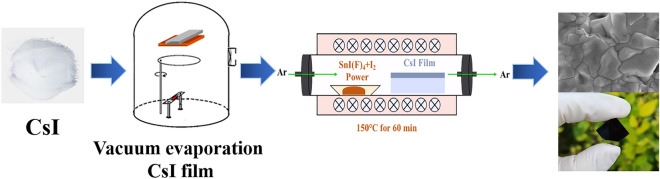
Schematic illustration for the preparation of Cs_2_SnI_6_ films.

The process of sample preparation was shown in Fig. 4. The glass slides were washed in acetone, ethanol, and deionized water for 15 min. The cleaning glass slide was transferred into a DM-450C vacuum system for CsI layer evaporation at a pressure of 3 × 10^−3^ Pa at room temperature. CsI powder was placed onto a tungsten (W) boat of size of 50 mm × 15 mm × 2 mm, which was 160 mm below the substrate. And then CsI films were placed in a quartz tube furnace with SnI_4_ and I_2_ powder in an alumina boat and annealed at 150 °C for 60 min (ramp rate: 4 °C/min) with Ar flow. After the reaction, the white translucent CsI films turned to mirror-like black Cs_2_SnI_6_ films. The experimental doping amount refers to the ratio of cell atoms, such as, F at% = 0, 25% and 50%, refer to Table 4.

**Table 4:** The original chemical composition of Cs_2_SnI_6_ films with F doped.

Sample	CsI (mmol)	SnI_4_ (mmol)	SnF_4_ (mmol)
Cs_2_SnI_6_	2.0	1.0	0
Cs_2_SnI_9/2_F_3/2_	2.0	0.75	0.25
Cs_2_SnI_3_F_3_	2.0	0.5	0.5

### Characterizations of Cs_2_SnI_6_

#### Structure and morphology characterization

X-ray diffraction (XRD) pattern were obtained by a X’Pert powder X-ray diffractometer in glancing angle scanning mode at 0.5° incident angle with Cu Ka X-ray (λ = 1.5418 Å) from 10° to 90° and analyzed by High-Score software. The top-view FESEM images of Cs_2_SnI_6_ films were observed by SIGMA HD Field Emission Scanning Electron Microscopy (FESEM), which was operated at 15 kV.

#### Optoelectronic characterization

The thicknesses of the films were measured by using a KLA Tencor Alpha-step D-100 type profilometer on a step created on the films by masking the glass substrates. The electrical properties of the films were measured by a HALL 8800 Hall Effect Measurement device. The samples were cut into 10 × 10 mm^2^ samples in a square shape. The measurement was carried out in four-point mode with gold electrodes by DC voltage in a 4000-gauss magnetic field at room temperature. A CARY 5000 UV–Vis–NIR spectrometer was used to measure the transmittance of the films over the wavelength range of 300 to 1500 nm.

### First-principles calculations

Calculations by the CASTEP module in Materials Studio were used to optimize the structure of Cs_2_SnI_6_ and Cs_2_SnI_6_ with F doped. After the structure was optimized, the (222) and (004) crystal plane formation energy of Cs_2_SnI_6_ phase were calculated. Then the effect of doped F^−^ on the change of preferred orientation is analyzed.

**Figure 5:**
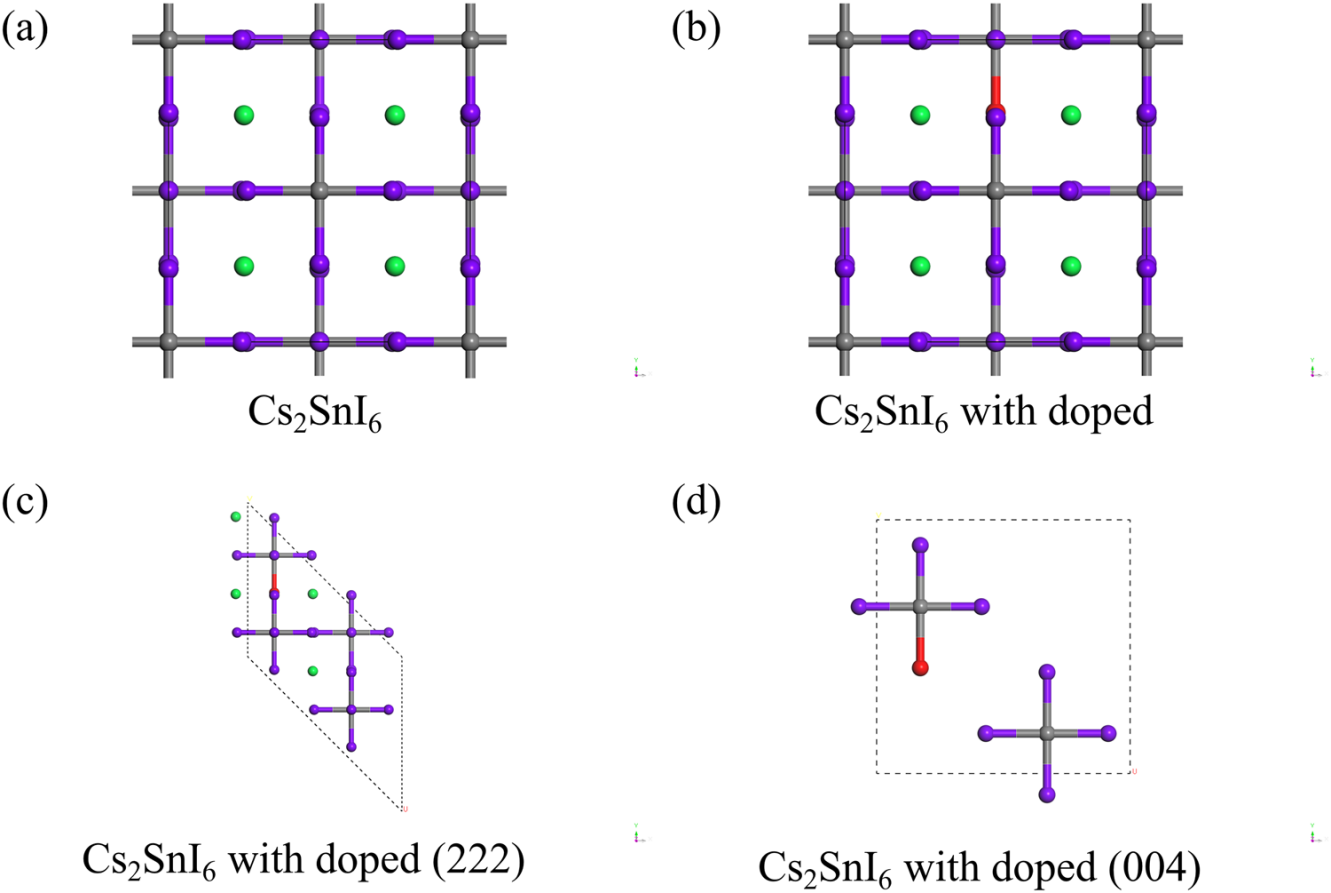
Calculation models: (**a**) Cs_2_SnI_6_ model; (**b**) Cs_2_SnI_6_ with doped model; (**c**) the cleaved (222) crystal plane model of Cs_2_SnI_6_ with doped; (**d**) the cleaved (004) crystal plane model of Cs_2_SnI_6_ with doped; Color code: Cesium, green; Tin, gray; Iodine, purple; Fluorine, red.

The Cs_2_SnI_6_ phase belongs to the room temperature phase stable structure, and its ground state three-dimensional model is a cubic crystal system. The structure of crystalline Cs_2_SnI_6_ (6 × 6 × 6) was obtained from the Materials Project database, with code number 27636^32^; the crystalline Cs_2_SnI_6_ had a FM-3 M space group symmetry. Combined with the doping ratio, a super cell with 18 atoms is established, and the valence electron configurations were Cs 6s^1^, Sn 5s^2^5p^2^, I 5s^2^5p^5^, and F 2p^5^. The super cell model is shown in Fig. 5a, and the F atom substitution doping model is shown in Fig. 5b. The (222) and (004) crystal plane models of Cs_2_SnI_6_ are shown in Fig. 5c,d, respectively.

## Supplementary Information

Supplementary Information.
