# Supplementary material for: The optoelectronic properties improvement of double perovskites Cs2SnI6 by anionic doping (F−)
Source: Sci Rep. 2022 Jan 18;12:935. doi: 10.1038/s41598-022-04960-2 (PMC8766447; doi:10.1038/s41598-022-04960-2)
Supplement: Supplementary file 1 — Supplementary Information. [file 41598_2022_4960_MOESM1_ESM.docx]

Supporting Information

The optoelectronic properties improvement of double perovskites Cs_2_SnI_6_ by anionic doping (F-)

[Junsheng Wu](https://orcid.org/0000-0003-2942-5830) ^1,2^, [Zhuo Zhao](https://orcid.org/0000-0003-1530-6750) ^1,2^ *, and [Yanwen Zhou](https://orcid.org/0000-0003-3636-0123) ^1,2^ *

^1^School of Chemical Engineering, University of Science and Technology LiaoNing, Anshan 114051, China.

^2^Research Institute of Surface Engineering, University of Science and Technology LiaoNing, Anshan 114051, China.

*****Correspondence: zhaozhuo@ustl.edu.cn; zhouyanwen1966@163.com.

### Experimental Details

### Characterizations of Cs_2_SnI_6_

### *Structure and Morphology Characterization*

X-ray photoelectron spectroscopy (XPS) data were obtained with an ESCALAB 250Xi electron spectrometer from Thermo Fischer using Al Ka radiations (hv=1486.6 eV). The base pressure was about 4×10^-9^ mbar. The working voltage was 14.6 kV, the filament current was 13.5 mA, and the signal was accumulated for 20 cycles. The test Passing-Energy was 20 eV, the step length was 0.1eV. The samples were cleaned by argon ion sputtering (3000 eV, 100 s) to remove the surface contaminants. The binding energies were referenced to the C 1s line at 284.8 eV from adventitious carbon.

### Figures


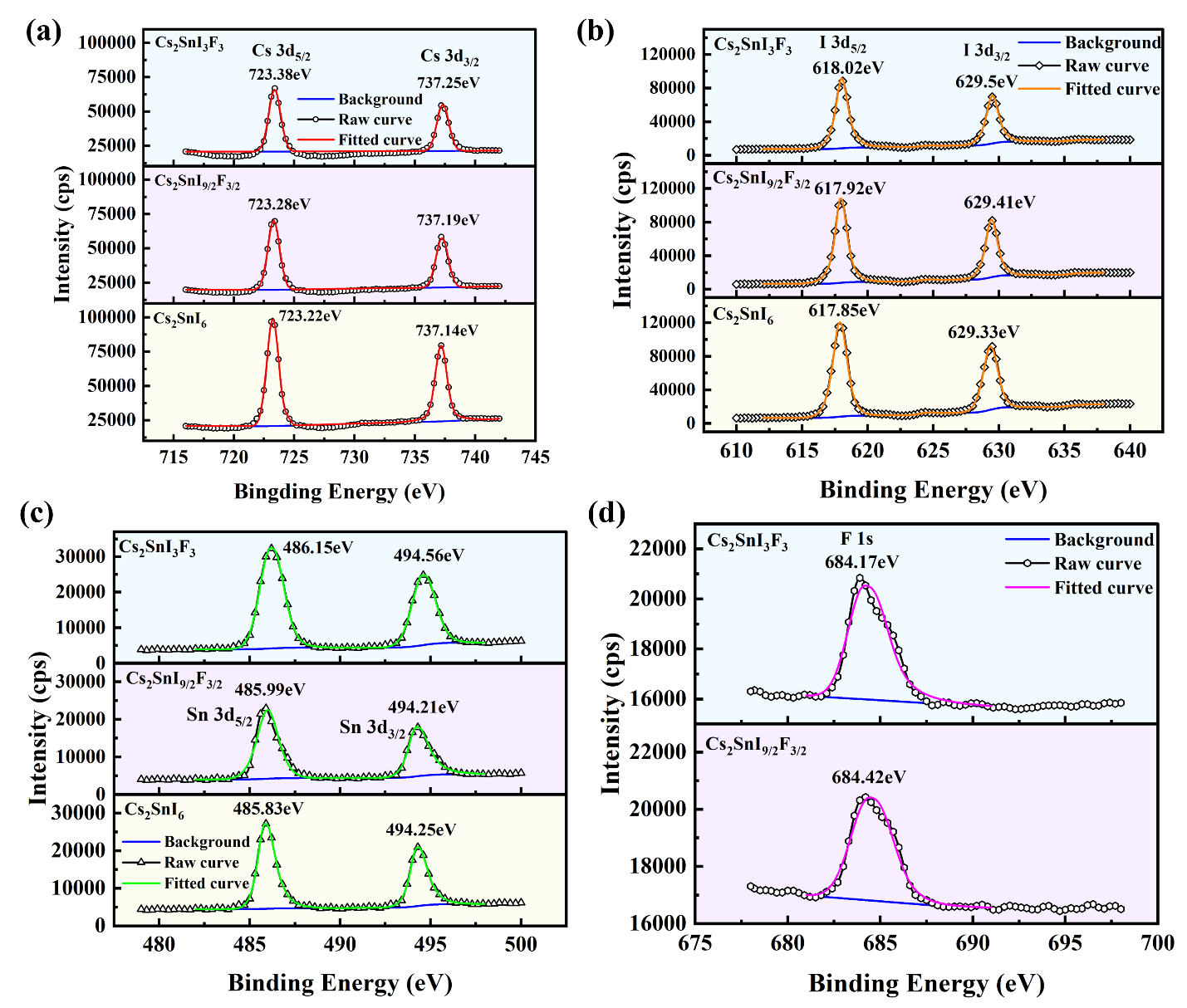


**Figure S1. The XPS spectra for (a) Cs 3d; (b) I 3d; (c) Sn 3d, and (d) F 1s elements of the films; Cs_2_SnI_6_ (Light yellow), Cs_2_SnI_9/2_F_3/2_ (Light pink), and Cs_2_SnI_3_F_3_ (Light blue) films respectively.**


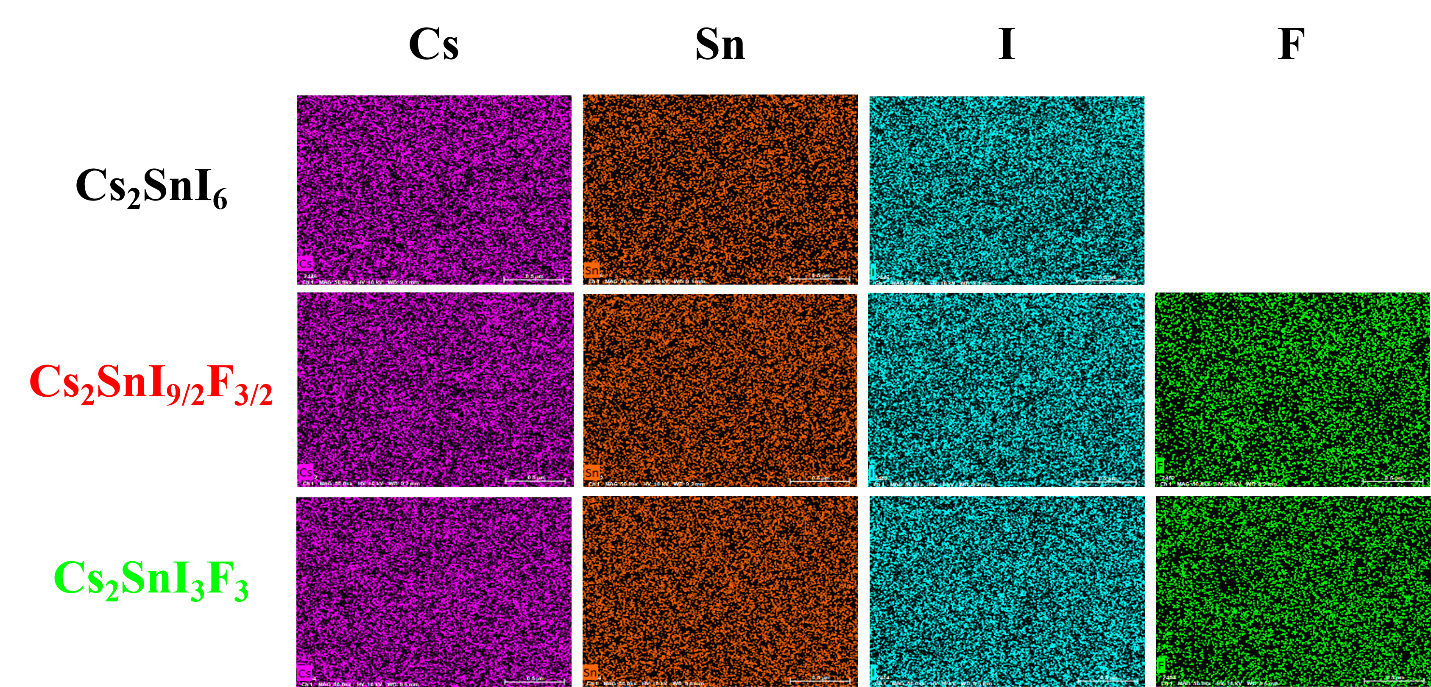


**Figure S2. EDX mapping for** **Cs_2_SnI_6_ films with F doped.**
